# An Observational Study to Evaluate the Risk Factors and Quality of Life in Individuals With Spinal Cord Injury and Pressure Injuries

**DOI:** 10.7759/cureus.98428

**Published:** 2025-12-04

**Authors:** Prasenjit Bhadra, Swapnil Sonune, Virendra Verma, John A Santoshi, Chethan Channaveera, Manal M Khan, Anyesha Saha, Niravkumar Ganpatram Joshi

**Affiliations:** 1 Physical Medicine and Rehabilitation, All India Institute of Medical Sciences, Bhopal, Bhopal, IND; 2 Physical Medicine and Rehabilitation, All India Institute of Medical Sciences, Nagpur, Nagpur, IND; 3 Orthopaedics, All India Institute of Medical Sciences, Bhopal, Bhopal, IND; 4 Physical Medicine and Rehabilitation, Atal Bihari Vajpayee Institute of Medical Sciences and Dr. Ram Manohar Lohia Hospital, New Delhi, IND; 5 Plastic Surgery, All India Institute of Medical Sciences, Bhopal, Bhopal, IND

**Keywords:** asia impairment scale, pressure injury, quality of life, risk factors, spinal cord injury

## Abstract

Background

Spinal cord injury (SCI) is a debilitating condition often associated with many complications, of which pressure injury (PI) is one of the most prevalent. Individuals with SCI are at an increased risk of developing PIs due to impaired protective sensations, dependence for mobility, and co-morbidities. Individuals with PIs often report an increase in morbidity, healthcare-related expenditure, and reduced quality of life (QoL). However, there is a paucity of data on PI-related risk factors and QoL outcomes in SCI individuals in developing countries like India. This study aimed to assess risk factors contributing to different stages of PI in individuals with SCI and to evaluate the impact of PI on their QoL.

Methodology

This was a cross-sectional observational study conducted over a period of 18 months at the All India Institute of Medical Sciences (AIIMS) Bhopal, India. A total of 134 individuals with SCI and PI were enrolled after considering all the inclusion and exclusion criteria. Data involving demographic profiling, physical examination, and hematological investigations were collected. Risk factors were assessed using the Braden Scale and Spinal Cord Injury Pressure Ulcer Scale (SCIPUS). Staging of PIs was done according to the Revised National Pressure Ulcer Advisory Panel Pressure Injury Staging System. Neurological level was classified using the American Spinal Injury Association (ASIA) Impairment Scale (AIS). QoL was determined using the World Health Organization Quality-of-Life Scale brief version (WHOQOL-BREF) questionnaire. Statistical analysis was done to assess the risk factors and their relation with SCI severity, PI stage, and QoL.

Results

The participants' mean age was 38.65 years and 76.87% were men. Labourers were the most often affected (35.82%). Most participants were paraplegic (64.93%), and trauma was the most common cause of SCI etiology (66.42%). Stage 4 PI was observed (35.24%) more often than other stages and was followed by Stage 2 PI (34.46%). Complete SCI (American Spinal Injury Association (ASIA) Impairment Scale (AIS) A) was strongly associated with higher-stage PI. Risk factors such as limited mobility, moisture, and friction/shear were significantly linked to higher PI stages. Bed-bound individuals were more prone to Stage 3, while stage 4 PI was predominant in wheelchair-bound individuals. Braden and SCIPUS scores showed decreasing risk for PI from AIS A to D. WHOQOL-BREF revealed that all domains of QoL were adversely affected, but social relationship was reported as a major concern in our participants. However, no significant association was found between stage of PI and QoL scores.

Conclusion

This study highlights the strong correlation between complete SCI and higher-stage of PIs, with modifiable risk factors like moisture and friction having a substantial impact. PI considerably impairs all QoL domains, particularly social relationships. These findings emphasise the need for targeted preventive strategies and standard protocols to arrest PI progression and improve QoL of individuals with SCI. This is among the first such studies in central India, offering valuable insights for future clinical and rehabilitative planning.

## Introduction

Spinal cord injury (SCI) describes damage to the spinal cord caused by trauma, disease, or degeneration. This damage can result in temporary or permanent changes in sensation, movement, strength, and function, below the site of injury. The annual incidence of such cases ranges between 2,50,000 and 5,00,000 globally [1]. SCI can be complicated by the presence of pressure injuries (PIs), autonomic dysreflexia, respiratory dysfunction, neuropathic pain, and neurogenic bowel and bladder. Notably, PIs represent one of the most frequent consequences of SCI, with a global prevalence of 30%-40% [2,3]. The pathophysiology of PI in individuals with SCI is intricate and can be attributed to many factors like motor, sensory, and autonomic impairments; restricted mobility and activity; nutrition and metabolic changes; and compromised psychological and behavioural status [4]. The interplay of these factors collectively contributes to a susceptible environment for the development of PI in the SCI population.

Pressure alone or exacerbated by shear stress can cause localized tissue damage, leading to the development of PI. Although pressure injuries frequently develop over bony prominences, they can also be related to medical or other devices [5]. It may or may not be painful and may appear as intact skin or an open ulcer. In cases of SCI, microclimate, nutrition, perfusion, comorbidities, and soft tissue status may also affect soft-tissue tolerance to pressure and shear. PIs have a huge impact on patients and the healthcare system, requiring extended hospital stays, multiple surgeries, and higher healthcare associated costs, with increased morbidity, and mortality. According to multiple studies, the mean length of hospital stay and healthcare cost for individuals with SCI having PIs is higher compared to those without [6,7]. Additionally, it affects the quality of life (QoL) of an individual [8]. 

The high prevalence of pressure injuries in individuals with SCI highlights the need for effective methods to prevent their occurrence and progression to higher stage. Healthcare providers must be watchful regarding the risk factors for PI development in this vulnerable population. The existing literature reveals a significant gap in our understanding of risk factors of PI and their impact on the QoL of individuals with SCI, particularly in developing countries like India. This lacuna highlights the imperative need of our study. This research addresses this particular crucial area of interest by investigating the relationship between common risk factors and the development, stage, and severity of PIs. It also focuses on their effect on quality of life of individuals with SCI. The findings from this study will contribute valuable insights to the existing data, potentially shaping more effective prevention strategies and improve outcomes in individuals with SCI, specially in resource-limited settings.

## Materials and methods

An observational cross-sectional study was undertaken at the All India Institute of Medical Sciences (AIIMS) Bhopal, a central India tertiary care facility. Ethical approval was granted by the Institutional Human Ethics Committee (IHEC-PGR/2021/PG/Jul/17), and written informed consent was provided by all participants in English or Hindi. The study was conducted over a period of 18 months (February 2022 to July 2023).

A total of 134 participants with SCI and PI admitted to the in-patient or outpatient departments were enrolled using a convenience sampling method after applying inclusion and exclusion criteria (Table 1).

**Table 1:** Inclusion and Exclusion Criteria

Inclusion Criteria	Exclusion Criteria
Individuals with spinal cord injury with pressure injuries	Congenital spinal dysraphism
Age above 18 years	Cognitive impairment
Any sex	Concomitant brain injury
All neurological levels of spinal cord injury	Other skeletal fractures
All stages of pressure injury	Any other condition that interferes with assessment
Duration of spinal cord injury less than five years	
Willingness to participate and provide written informed consent	

Data regarding demographic characteristics, injury-related parameters, neurological status, and pressure injury assessments were systematically collected using standardized case record forms. The dataset was subsequently analyzed using descriptive statistical techniques using IBM SPSS Statistics software version 25.0 (IBM Corp., Armonk, NY).

The neurological level and severity of spinal cord injury were established according to the American Spinal Injury Association (ASIA) Impairment Scale (AIS), in conformity with the International Standards for Neurological Classification of Spinal Cord Injury and International Spinal Cord Society (ISCOS) guidelines [9]. PI was staged according to the Revised National Pressure Ulcer Advisory Panel Pressure Injury Staging System [10]. The risk factors for PI were assessed with both the Braden Scale and the Spinal Cord Injury Pressure Ulcer Scale (SCIPUS) [11,12]. Health-related quality of life was measured by the World Health Organization Quality of Life Instrument - brief version (WHOQOL-BREF) [13].

## Results

Demographic profile

This study examined demographic data (Table 2) and analyzed the relationship between risk factors of PI, SCI severity, and participants' QoL. The cohort comprised 134 individuals, predominantly men (76.87%), of age 19-77 years (mean: 38.65 years). The 18-30 age group was most represented. Literacy varied, with a small fraction (11.94%) reporting no formal schooling. Occupationally, laborers constituted the largest group (46.27%) among those with PI and SCI. Paraplegia was the predominant condition (64.93%), with C4 and D12/L1 being the most frequent neurological injury levels for tetraplegics and paraplegics, respectively. Traumatic injuries accounted for the majority of cases (66.42%), with falls from height being the primary cause (26.12%).

**Table 2:** Demographic data distribution

Demographic categories	Frequency	Percentage
Age		
≤30	54	40.30%
31-40	24	17.91%
41-50	24	17.91%
51-60	21	15.67%
61-70	7	5.22%
71-80	4	2.99%
Gender		
Female	31	23.13%
Male	103	76.87%
Education		
No schooling	16	11.94%
Primary	15	11.19%
Secondary	31	23.13%
Higher secondary	49	36.57%
Graduate and postgraduate	23	17.16%
Occupation		
Employed	24	17.91%
Unemployed	11	8.21%
Student	15	11.19%
Labour	62	46.27%
Homemaker	22	16.42%
Neurological level of injury		
Cervical	47	35.08%
Upper thoracic	14	10.45%
Lower thoracic	45	33.58%
Lumbar	28	20.89%
Mode of Injury		
Traumatic	89	66.42%
Non-Traumatic	45	33.58%

Association of risk factors with pressure injury

The risk factors associated with the development and severity of pressure injuries were evaluated using the Braden and SCIPUS scales. The findings are summarized in Tables 3, 4.

**Table 3:** Association of Braden Scale risk factors with stage of pressure injury *Chi-square test, **Fisher's Exact test; threshold for statistical significance: p<0.05.

Braden scale (Risk factor)	Stage 1 (n=8)	Stage 2 (n=78)	Stage 3 (n=43)	Stage 4 (n=80)	Unstageable pressure injury (n=15)	Deep-tissue pressure injury (n=3)	Total	P value
Sensory perception								
Completely limited	3 (37.50%)	44 (56.41%)	28 (65.12%)	49 (61.25%)	8 (53.33%)	2 (66.67%)	134 (59.03%)	0.381^*^
Very limited	1 (12.50%)	17 (21.79%)	9 (20.93%)	20 (25%)	6 (40%)	1 (33.33%)	54 (23.79%)	
Slightly limited	4 (50%)	17 (21.79%)	6 (13.95%)	11 (13.75%)	1 (6.67%)	0 (0%)	39 (17.18%)	
Activity								
Bedfast	3 (37.50%)	26 (33.33%)	22 (51.16%)	33 (41.25%)	9 (60%)	1 (33.33%)	94 (41.41%)	0.02^*^
Chairfast	5 (62.50%)	40 (51.28%)	18 (41.86%)	46 (57.50%)	6 (40%)	1 (33.33%)	116 (51.10%)	
Walks occasionally	0 (0%)	12 (15.38%)	3 (6.98%)	1 (1.25%)	0 (0%)	1 (33.33%)	17 (7.49%)	
Moisture								
Constantly moist	6 (75%)	40 (51.28%)	25 (58.14%)	49 (61.25%)	13 (86.67%)	2 (66.67%)	135 (59.47%)	<.0001^*^
Often moist	0 (0%)	17 (21.79%)	4 (9.30%)	12 (15%)	1 (6.67%)	0 (0%)	34 (14.98%)	
Occasionally moist	0 (0%)	6 (7.69%)	2 (4.65%)	0 (0%)	0 (0%)	1 (33.33%)	9 (3.96%)	
Rarely moist	2 (25%)	15 (19.23%)	12 (27.91%)	19 (23.75%)	1 (6.67%)	0 (0%)	49 (21.59%)	
Mobility								
Completely immobile	0 (0%)	17 (21.79%)	13 (30.23%)	19 (23.75%)	4 (26.67%)	0 (0%)	53 (23.35%)	0.059^*^
Very limited	8 (100%)	51 (65.38%)	27 (62.79%)	60 (75%)	11 (73.33%)	2 (66.67%)	159 (70.04%)	
Slightly limited	0 (0%)	10 (12.82%)	3 (6.98%)	1 (1.25%)	0 (0%)	1 (33.33%)	15 (6.61%)	
Nutrition								
Very poor	0 (0%)	1 (1.28%)	1 (2.33%)	1 (1.25%)	1 (6.67%)	0 (0%)	4 (1.76%)	0.084^**^
Probably inadequate	8 (100%)	74 (94.87%)	42 (97.67%)	79 (98.75%)	14 (93.33%)	2 (66.67%)	219 (96.48%)	
Adequate	0 (0%)	3 (3.85%)	0 (0%)	0 (0%)	0 (0%)	1 (33.33%)	4 (1.76%)	
Friction/shear								
Problem	6 (75%)	50 (64.10%)	39 (90.70%)	73 (91.25%)	15 (100%)	2 (66.67%)	185 (81.50%)	<.0001^**^
Potential problem	2 (25%)	28 (35.90%)	4 (9.30%)	7 (8.75%)	0 (0%)	1 (33.33%)	42 (18.50%)	

**Table 4:** Association of SCIPUS risk factors with stage of pressure injury SCIPUS: Spinal Cord Injury Pressure Ulcer Scale; SCI: spinal cord injury; ABN: abnormal. *Chi-square test, **Fisher's Exact test, threshold for statistical significance: p<0.05.

SCIPUS (Risk factor)	Stage 1 (n=8)	Stage 2 (n=78)	Stage 3 (n=43)	Stage 4 (n=80)	Unstageable pressure injury (n=15)	Deep-tissue pressure injury(n=3)	Total	P value
Mobility								
Limited	8 (100%)	61 (78.21%)	30 (69.77%)	61 (76.25%)	11 (73.33%)	3 (100%)	174 (76.65%)	0.535^*^
Immobile	0 (0%)	17 (21.79%)	13 (30.23%)	19 (23.75%)	4 (26.67%)	0 (0%)	53 (23.35%)	
Level of activity								
Ambulatory	0 (0%)	12 (15.38%)	3 (6.98%)	1 (1.25%)	0 (0%)	1 (33.33%)	17 (7.49%)	0.02^*^
Wheelchair	5 (62.50%)	40 (51.28%)	18 (41.86%)	46 (57.50%)	6 (40%)	1 (33.33%)	116 (51.10%)	
Bed	3 (37.50%)	26 (33.33%)	22 (51.16%)	33 (41.25%)	9 (60%)	1 (33.33%)	94 (41.41%)	
Age (years)								
≤34	5 (62.50%)	35 (44.87%)	26 (60.47%)	41 (51.25%)	7 (46.67%)	2 (66.67%)	116 (51.10%)	0.907^*^
35 to 64	3 (37.50%)	37 (47.44%)	15 (34.88%)	33 (41.25%)	8 (53.33%)	1 (33.33%)	97 (42.73%)	
≥65	0 (0%)	6 (7.69%)	2 (4.65%)	6 (7.50%)	0 (0%)	0 (0%)	14 (6.17%)	
Tobacco use/smoking								
Never	5 (62.50%)	52 (66.67%)	31 (72.09%)	56 (70%)	14 (93.33%)	3 (100%)	161 (70.93%)	0.791^*^
Former	3 (37.50%)	21 (26.92%)	9 (20.93%)	18 (22.50%)	1 (6.67%)	0 (0%)	52 (22.91%)	
Current	0 (0%)	5 (6.41%)	3 (6.98%)	6 (7.50%)	0 (0%)	0 (0%)	14 (6.17%)	
Complete SCI	4 (50%)	38 (48.72%)	31 (72.09%)	58 (72.50%)	9 (60%)	1 (33.33%)	141 (62.11%)	0.016^*^
Urine incontinence/constantly moist	6 (75%)	63 (80.77%)	31 (72.09%)	61 (76.25%)	14 (93.33%)	3 (100%)	178 (78.41%)	0.554^*^
Autonomic dysreflexia/severe spasticity	0 (0%)	23 (29.49%)	9 (20.93%)	17 (21.25%)	4 (26.67%)	1 (33.33%)	54 (23.79%)	0.431^*^
Pulmonary disease	0 (0%)	2 (2.56%)	0 (0%)	0 (0%)	0 (0%)	0 (0%)	2 (0.88%)	0.369^**^
Cardiac disease/ABN (EKG)	0 (0%)	4 (5.13%)	1 (2.33%)	3 (3.75%)	0 (0%)	0 (0%)	8 (3.52%)	0.93^**^
Diabetes/glucose≥110 mg/dL	1 (12.50%)	8 (10.26%)	3 (6.98%)	7 (8.75%)	0 (0%)	0 (0%)	19 (8.37%)	0.804^**^
Renal disease	0 (0%)	0 (0%)	0 (0%)	0 (0%)	0 (0%)	0 (0%)	0 (0%)	NA
Impaired cognitive function	0 (0%)	0 (0%)	0 (0%)	0 (0%)	0 (0%)	0 (0%)	0 (0%)	NA
In a nursing home/hospital	8 (100%)	78 (100%)	43 (100%)	80 (100%)	15 (100%)	3 (100%)	227 (100%)	NA
Albumin<3.4 g/dL/Total protein<6.4 g/dL	3 (37.50%)	29 (37.18%)	14 (32.56%)	30 (37.50%)	9 (60%)	1 (33.33%)	86 (37.89%)	0.59^*^
Hemoglobin<12 g/dL/Hematocrit<36%	3 (37.50%)	34 (43.59%)	18 (41.86%)	49 (61.25%)	11 (73.33%)	1 (33.33%)	116 (51.10%)	0.052^*^

Complete spinal cord injuries (AIS A) were present in 55.22% of the participants, and stage 4 pressure injuries were the most frequent (35.24%), followed closely by stage 2 injuries (34.46%). Limited sensory perception was observed across all stages of PI.

Participants classified as “Bedfast” were more likely to have advanced PIs (stage 3 or higher), whereas “Chairfast” individuals showed a higher prevalence of stage 4 PIs. Similarly, severely restricted mobility (“Very limited”) correlated with higher-stage PIs, although the association was weak.

Moisture-related factors, such as constant moisture or friction/shear issues, were significantly associated with advanced pressure injury stages. In contrast, factors such as age, tobacco use, nutritional status, and other medical conditions showed no significant association with the stage of PI.

**Figure 1:**
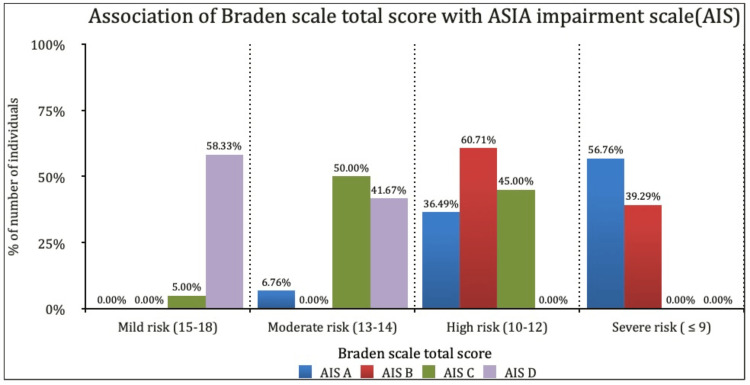
Association of Braden Scale total score with ASIA Impairment Scale (AIS) ASIA: American Spinal Injury Association.

**Figure 2:**
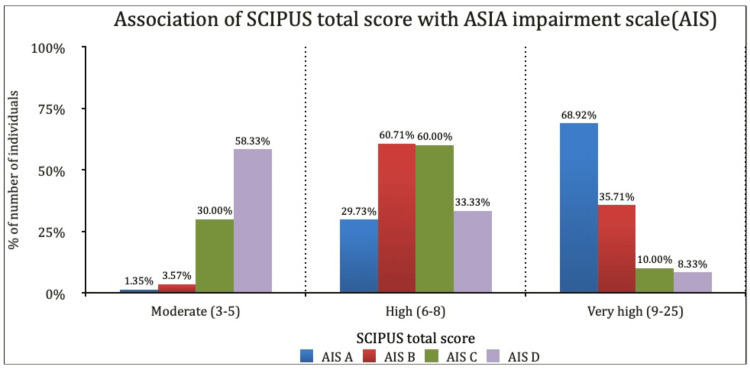
Association of SCIPUS total score with ASIA Impairment Scale (AIS) SCIPUS: Spinal Cord Injury Pressure Ulcer Scale; ASIA: American Spinal Injury Association.

The total risk scores from the Braden and SCIPUS scales demonstrated a significant decreasing trend from complete (AIS A) to incomplete injuries (AIS D), as depicted in Figures 1, 2.

Association of quality of life with pressure injury

The impact of PIs on the quality of life of individuals with SCI was assessed across four domains-physical health, psychological well-being, social relationships, and environmental factors-using the WHOQOL-BREF tool. The results are shown in Table 5.

**Table 5:** Association of WHOQOL-BREF with stage of pressure injury **ANOVA test, threshold for statistical significance: p<0.05. WHOQOL-BREF: World Health Organization Quality of Life Scale - brief version; ANOVA: analysis of variance

WHOQOL-BREF	Stage 1 (n=8)	Stage 2 (n=78)	Stage 3 (n=43)	Stage 4 (n=80)	Unstageable pressure injury (n=15)	Deep tissue pressure injury (n=3)	Total	P value
Physical health domain								
Mean ± SD	15.38±2.45	14.95±2.91	15.07±2.79	16.1±4.02	14.67±3.56	19±8.89	15.43±3.48	0.116^**^
Psychological domain								
Mean ± SD	17.88±3.64	17.88±3.24	18.28±2.83	18.16±3	17.4±2.13	20±4.36	18.05±3.03	0.773^**^
Social relationship domain								
Mean ± SD	9.75±1.58	10.81±1.6	10.88±1.48	10.69±1.49	9.8±1.57	11.33±2.08	10.68±1.56	0.091^**^
Environment domain								
Mean ± SD	26.88±4.85	28.47±4.53	28.65±2.79	27.78±4.51	26.47±7.31	25.33±3.21	28.03±4.49	0.385^**^

All areas of QoL were negatively impacted by PIs. The social relationship domain showed the most significant decline, with an average score as low as 11. This suggests a lack of support from the family and society and dissatisfaction with interpersonal relationships. The physical health domain was also significantly affected, with a mean score of 15, indicating functional limitations and inadequate pain management. In contrast, the environmental domain had the least impact, scoring a mean of 29, which indicates relatively better access to medical as well as non-medical services and resources.

Despite these trends, the statistical analysis did not show a significant link between the stage of PI and QoL scores in any of the domains (physical, psychological, social relationship, and environmental). This means that PIs, regardless of stage, invariably impair QoL in individuals with SCI without stage-dependent influence.

These findings align with the clinical and demographic characteristics discussed above. Limited mobility, sensory impairment, and risks such as friction and moisture were linked to higher-stage pressure injuries (Tables 3, 4). The consistently negative impact of pressure injuries on QoL, observed here, highlights the widespread burden across all severity levels.

## Discussion

This study examines the demographics, clinical features, and risk factors of developing PIs in people with SCI. It also looks at their QoL. The results are similar to previous research but add new information pertaining to central India.

Demographic characteristics

Most people in this group were young adults aged 18-30 years, which matches earlier studies showing this age group is especially at risk for PIs after SCI [14]. Young adults often face more traumatic injuries because of their jobs and adventure-seeking behaviour.

There were more men than women in the study, with men making up about 77% of the participants. This supports earlier research showing men are more likely to have PIs [14-16], likely because they experience more traumatic spinal injuries from work as well as accidents.

Participants had different levels of education. About 37% had completed higher secondary school, 17% were pursuing higher education, and nearly 12% had no formal education. While people with more education are usually thought to have better health habits, our results show that many with higher education still had PIs. Other studies found similar patterns, but did not show a strong link between education level and risk [17]. This could be because people with less education may have been missed due to hospital-based convenience sampling, possibly due to challenges in accessing healthcare.

Clinical characteristics and risk factors

Paraplegia (64.93%) was more prevalent than tetraplegia in this cohort, similar to the findings by many other researchers [18-20], although differing from the study by Grigorian et al., which reported a higher incidence of cervical-level injuries [21]. For those with paraplegia, the D12 and L1 neurological levels were more common, while C4 was predominant NLI in tetraplegia. Even though people with paraplegia have better upper-body movement, they may still get PIs because of extra strain during transfers and prolonged or poor seating.

Traumatic spinal injuries made up 66.42% of cases, with falls from heights (26.12%) being the leading cause, followed by road traffic accidents (16.42%). This trend matches results from Taghipoor et al., who found that 71.8% of individuals with SCI and PIs had traumatic origins [22]. Among non-traumatic cases, Pott's spine was the main cause, accounting for 12.69% of instances. Both traumatic injuries and infections like Pott's spine often require extended immobilization or surgery, increasing the risk of PIs. 

Most participants across all stages of PIs had reduced sensory perception. Sensory deficits are a known risk factor because they hinder the ability to feel the discomfort and change positions, which leads to prolonged pressure on vulnerable tissues [23]. Similarly, immobility proved to be a critical factor. Individuals who were “bedfast” were more likely to have Stage 3 injuries, and those who were “chair-fast” were more prone to Stage 4 injuries. The lack of padding and increased friction in chairs likely contributed to this heightened risk, echoing the findings of Heather Orsted et al. [23].

Immobility, moisture, and friction/shear were clearly linked to the advanced stages of PIs. People with “very limited” mobility had a higher occurrence of higher-stage PIs. The constant moisture as a result of incontinence, coupled with friction and shear problems, exacerbated tissue breakdown. This shows the heightened risk of those with reduced mobility and long-term exposure to moisture, which concurs with the known pathophysiology of damage.

Surprisingly, no direct correlations with PI severity were made with such factors as age, tobacco smoking, nutrition, muscle spasms, cardiovascular or pulmonary disease, anemia, or renal disease. This would suggest that, although these factors can influence general health status, they do not play a determining role in the severity of PI in this population.

Complete spinal cord injury (AIS A) was noted in 55.22% of the participants and was significantly linked to a higher risk of PIs, reinforcing earlier findings by Irgens et al. [24]. The severity of the injury, as indicated by the AIS, corresponded with higher scores on the Braden and SCIPUS risk assessment tools. These connections confirm that individuals with complete injuries face greater mobility challenges, weight-shifting difficulties, and self-care issues, making them more susceptible to severe PIs.

The overall risk factors assessment according to our results of the study is summarised in Table 6.

**Table 6:** Summary of Risk Factors Associated with Pressure Injury in SCI Patients AIS: American Spinal Injury Association (ASIA) Impairment Scale; SCI: spinal cord injury.

Risk Factors	Association with Pressure Injury	Findings from the Study
Sensory Perception (Completely/Very limited)	Directly Associated	Limited sensation increased risk of PI due to inability to detect pressure and discomfort, leading to prolonged pressure exposure.
Mobility (Bedfast, Chair-fast)	Directly Associated	Immobility correlated with higher stages of PI; chair-fast individuals had more advanced injuries due to friction and less cushioning.
Friction/Shear	Directly Associated	High prevalence (76.12%) of friction and shear problems; significantly related to higher-stage injuries due to tissue tearing and pressure forces.
Moisture (Constantly moist)	Directly Associated	Moisture from incontinence weakened skin integrity, contributing to higher-stage PI.
Severity of SCI (Complete injury, AIS A)	Directly Associated	Complete injuries had a greater risk; decreased mobility and reliance on caregivers led to higher incidence of advanced PI stages.
Use of Preventive Measures	Relatively Associated	Not directly measured but inferred; proper skin care, seating, and repositioning may mitigate risk.
Age	Not Significantly Associated	No significant correlation found between age and PI stage, despite a higher number of younger adults affected.
Tobacco Use/Smoking	Not Significantly Associated	No statistically significant link with PI severity.
Nutrition (Hypoproteinemia, Anemia)	Not Significantly Associated	No significant association, possibly due to uniformity across participants or lack of severe deficiencies.
Spasticity/Autonomic Dysreflexia	Not Significantly Associated	No significant correlation observed with PI stage.
Pulmonary/Cardiac/Renal Diseases	Not Significantly Associated	Comorbidities did not show significant association with PI stage in this sample.

Quality of life

Quality of life, as measured by the WHOQOL-BREF, was compromised in all dimensions: physical, psychological, social relationships, and environment. Social relationships were most affected, with high levels of social isolation and low levels of personal engagement. Physical well-being also suffered, with pain, functional impairment, and mobility problems contributing to lower well-being.

In contrast to research such as that conducted by Mota Dalete et al., who identified increased dissatisfaction in the physical domain [25], our findings report the more extensive psychosocial issues of those with SCI and PIs in India. Notably, we did not identify a significant relationship between PI stage and quality of life scores, indicating that lower-stage injuries can have comparable adverse impacts on social interaction and mental well-being.

A number of socio-cultural variables can help account for these findings. People with SCI in India tend to face stigma and myths, which limit their integration into society and access to services. Inadequate infrastructure and outdated beliefs compound these issues. Family relationships are the key to caregiving but place a strain on relationships and alter family roles. Economic disparities exacerbate these issues, hindering access to healthcare and rehabilitation services.

Limitations

This was a descriptive study based on convenience sampling, reporting associations but not causality. The sample was unlikely to represent all SCI patients, and confounding variables were not controlled for in preventive interventions, and long-term consequences were not evaluated.

Future research prospects

Subsequent research needs to employ longitudinal designs based on more representative samples in order to explore causal effects and the efficacy of prevention interventions. Studies involving multi-center information, socioeconomic variables, and technological resources can further enrich the recognition and control of PIs in SCI.

## Conclusions

The findings of this study strongly indicate that certain risk factors are strongly associated with PI development and severity for patients with SCI. Limited sensory perception, immobility (bedfast or chair-fast), friction and shear forces, incontinence moisture, and the severity of the spinal injury (complete injury, AIS A) are significant factors that lead to severe PIs. On the other hand, age, tobacco smoking, dietary inadequacies, muscle cramps, autonomic dysfunction, and other systemic diseases failed to show significant associations with PI severity, but may well still be influencing overall well-being.

The results emphasise the necessity of interventions aimed at increasing mobility, skin care, moisture management, and seating ergonomics, particularly among those with complete injury and reduced sensation. Intervening on these modifiable risk factors through education, the use of assistive devices, and caregivers' training may be essential to diminish the pressure injury burden in individuals with SCI.
